# Cerebellum and basal ganglia connectivity in isolated REM sleep behaviour disorder and Parkinson’s disease: an exploratory study

**DOI:** 10.1007/s11682-024-00939-x

**Published:** 2024-09-25

**Authors:** Michael J. Firbank, Jacopo Pasquini, Laura Best, Victoria Foster, Hilmar P. Sigurdsson, Kirstie N. Anderson, George Petrides, David J. Brooks, Nicola Pavese

**Affiliations:** 1 Translational and Clinical Research Institute, Newcastle University, Campus for Ageing and Vitality, Newcastle upon Tyne, NE4 5PL UK; 2 Department of Clinical and Experimental Medicine, University of Pisa, Pisa, Italy; 3 Nuclear Medicine Department, Newcastle upon Tyne Hospitals NHS Foundation Trust, Newcastle upon Tyne, UK; 4 Department of Nuclear Medicine & PET, Aarhus University Hospital, Aarhus, Denmark

**Keywords:** REM sleep behaviour disorder, Connectivity, Resting state fMRI, Cerebellum, Thalamus

## Abstract

REM sleep behaviour disorder (RBD) is a parasomnia characterised by dream-enacting behaviour with loss of muscle atonia during REM sleep and is a prodromal feature of α-synucleinopathies like Parkinson’s disease, dementia with Lewy bodies, and multiple system atrophy. Although cortical-to-subcortical connectivity is well-studied in RBD, cerebellar and subcortical nuclei reciprocal connectivity is less established. Nonetheless, it could be relevant since RBD pathology involves brainstem structures with an ascending gradient. In this study, we utilised resting-state functional MRI to investigate 13 people with isolated RBD (iRBD), 17 with Parkinson’s disease and 16 healthy controls. We investigated the connectivity between the basal ganglia, thalamus and regions of the cerebellum. The cerebellum was segmented using a functional atlas, defined by a resting-state network-based parcellation, rather than an anatomical one. Controlling for age, we found a significant group difference (F_4,82_ = 5.47, p_FDR_ = 0.017) in cerebellar-thalamic connectivity, with iRBD significantly lower compared to both control and Parkinson’s disease. Specifically, cerebellar areas involved in this connectivity reduction were related to the default mode, language and fronto-parietal resting-state networks. Our findings show functional connectivity abnormalities in subcortical structures that are specific to iRBD and may be relevant from a pathophysiological standpoint. Further studies are needed to investigate how connectivity changes progress over time and whether specific changes predict disease course or phenoconversion.

**Supplementary Information:**

The online version contains supplementary material available at 10.1007/s11682-024-00939-x.

## Introduction

REM sleep behaviour disorder (RBD) is a REM parasomnia characterised by vivid dreaming and failure to develop muscle atonia, leading to sometimes violent limb movements and vocalisations. (American Academy of Sleep Medicine, 2014) The condition is frequently present in disorders associated with abnormal α-synuclein aggregation - Parkinson’s disease (PD), dementia with Lewy bodies (DLB) and multiple system atrophy (MSA) (Hu, 2020). People with isolated RBD (iRBD), i.e. RBD in the absence of another neurological disorder, frequently go on to develop one of these conditions, with a multicentre study finding a conversion rate of 74% over 12 years. (Postuma et al., 2019) Therefore, iRBD is now considered to be a prodromal phenotype of PD, and subclinical alterations in the dopaminergic, noradrenergic and cholinergic systems (Stokholm et al., 2020; Knudsen et al., 2018) have been observed before the development of overt motor or cognitive symptoms. Furthermore, iRBD often displays an ascending pathological gradient that involves the peripheral autonomic system and brainstem structures, including those involved in REM sleep atonia generation. (Horsager et al., 2020).

Anatomical connections between the brainstem nuclei and the cerebellum, thalamus and basal ganglia (Liebe et al., 2020; Muthasamy et al., 2007; García-Gomar et al., 2022) have been documented, as well as connections between these last three structures. (Habas, 2019; Bostan & Strick, 2010; Alexander et al., 1986) The cerebellum projects to the motor cortex via the ventral intermediate and ventrolateral nuclei of the thalamus, (Zhong et al., 2022; Spampinato, 2023) and influences the motor cortex during sleep. (Xu et al., 2021) The cerebellum also connects to the centromedian thalamic nucleus (Habas, 2019) which is involved in sleep / wake transitions. A number of studies have investigated iRBD with resting state fMRI and have reported changes in functional connectivity between the cortex and the putamen, (Dayan & Browner, 2017; Ellmore et al., 2013; Yamada et al., 2019) thalamus (Byun et al., 2020) and cerebellum (Yamada et al., 2019; Li et al., 2021; Liu et al., 2021). However, studies that investigate the functional connections between all these subcortical structures are lacking, although such investigations are important to understand the pathophysiological differences between iRBD and PD and the mechanisms of both motor and non-motor symptoms.

To investigate the cerebellar functional connections, in this study we used the recently developed fMRI-based cerebellar parcellation (Ji et al., 2019) of cerebellar function rather than the classical anatomical segmentation (King et al., 2019; Ji et al., 2019) as done in previous studies. (Yamada et al., 2019) This segmentation identifies ten cerebellar regions relating to well-known cortical networks (e.g. default mode, somatomotor, frontoparietal networks) and allows the investigation of connectivity measures between these functional areas and subcortical nuclei.

The main purpose of the study was to investigate connectivity abnormalities in the iRBD and PD groups between the basal ganglia, brainstem, cerebellum, and thalamus and the associations with symptom burden.

## Methods

### Participants

A total of 19 people with PD and 14 with iRBD aged between 45 and 80 years were recruited between June 2019 and September 2021 from our Movement Disorder Clinics and the Sleep Unit at Newcastle Hospitals NHS Trust, Newcastle upon Tyne, UK.

PD diagnosis was made according to the MDS PD Criteria (Postuma et al., 2015) by a movement disorder specialist while iRBD patients had polysomnography-confirmed RBD according to established criteria. (American Academy of Sleep Medicine, 2014) Before inclusion, iRBD patients underwent a full clinical history and examination to exclude a neurological condition. None of the iRBD patients had motor or cognitive symptoms.

We also recruited 18 people of similar age with no neurological signs or symptoms as healthy controls (HC). All study participants had full mental capacity and gave written informed consent. Healthy controls were screened for absence of RBD symptoms with the RBD screening questionnaire (RBDSQ) (Stiasny-Kolster et al., 2007) and with a comprehensive clinical history of the individual, taken by interview with the individual and their bed partner.

Exclusion criteria for PD, iRBD patients and HC included (a) diagnosis of other forms of atypical parkinsonism, including DLB, MSA, progressive supranuclear palsy, corticobasal syndrome or drug-induced parkinsonism; (b) significant cognitive impairment at presentation (defined as MMSE < 24) or meeting DSM V criteria for major neurocognitive disorder; (c) lack of capacity to consent to be involved in the study; (d) contraindications to MRI, including claustrophobia; (e) severe co-morbid medical illness as determined by the investigator. For iRBD patients and HC, people suspected of having parkinsonism prior to the onset of the study were excluded.

Ethical approval was granted by the London-Surrey Research Ethics Committee Research Ethics Committee (18/LO/2123).

### Assessment

The clinical features of REM Sleep Behaviour disorder were assessed using the RBD screening questionnaire (RBDSQ). (Stiasny-Kolster et al., 2007) The MDS-Unified Parkinson’s Disease Rating Scale (Goetz et al., 2008) was used to assess motor and non-motor features of PD. Global cognition was assessed with the Montreal Cognitive Assessment (MoCA) (Nasreddine et al., 2005) and the mini mental state exam (MMSE). For PD patients, all clinical and imaging assessments were performed in an ON state following their normal doses of dopamine replacement therapy.

### Image acquisition

Participants were scanned on a 3T PET-MR scanner (Signa, GE Medical Systems, USA) with a 32-channel head coil. PD participants were scanned ON medication. Images acquired included whole brain 3D structural scan Fast Spoiled Gradient Echo (FSPGR) with repetition time (TR) = 7ms, echo time (TE) = 3ms, flip angle = 11°, 1 mm cubic voxels, parallel acceleration factor (ASSET) = 2; Resting state fMRI with gradient echo – echo planar imaging (GE-EPI) with TR = 2080 ms, TE = 30 ms, flip angle = 90°, parallel acceleration factor (ASSET) = 2, number slices = 36, slice thickness = 3.5 mm (+ 0.7 mm gap), field of view = 192 × 192 mm, matrix size = 64 × 64, 285 volumes (plus 3 dummy scans) acquisition time = 10 min. We also acquired a pair of spin echo (SE) EPI images with the same geometry with phase encoding in the opposite direction for use in distortion correction.

### Image processing

Structural images were processed with SPM (version 7771) and the CAT12 toolbox (version 1907 https://neuro-jena.github.io/cat/*)* using default values to segment images into grey and white matter and CSF, and spatially normalise to the CAT12 standard template. Thalamic and basal ganglia regions of interest on the caudate, putamen, pallidum and thalamus were taken from the neuromorphometrics atlas, while the substantia nigra was from the AAL3 atlas (Rolls et al., 2020), (both provided with CAT12) and transformed (using the inverse of the spatial normalisation) to the individual subject for use in fMRI analysis. A skull-stripped version of each subject’s structural scan was generated using a mask from the default CAT12 output binary tissue classification image.

We used the SUIT toolbox for SPM (https://www.diedrichsenlab.org/imaging/suit.htm) to define functional regions of the cerebellum. We created a cerebellum and brainstem mask based on the default mask in SUIT, but matching the CAT12 template (MNI152NLin2009cAsym space). This was then transformed using the CAT12 normalisation function to the space of each subject, and manually edited to remove non cerebellar tissue. We then used the SUIT normalize routine to calculate the transform of the CAT12 segmentations to the SUIT cerebellum template. The 10-region cerebellar resting-state atlas (Ji et al., 2019) as supplied in SUIT was then transformed back to each subject, and used in the Conn toolbox to extract the time course from the native space unsmoothed resting state scans. The registration of all subjects’ structural scans to the CAT12 and cerebellar templates was visually checked, and the position of the cerebellar, thalamic and basal ganglia ROIs were checked on each participant’s fMRI scan. Supplementary figures S1 and S2 show the location of the ROIs on a typical iRBD participant.

The Conn toolbox (version CONN21.a https://web.conn-toolbox.org/home) was then used for preprocessing fMRI and the connectivity analysis. The fMRI scans were motion corrected, unwarped, aligned to the skull stripped structural scan, denoised and outlier scans identified. Confounds in the connectivity analysis were the default aCompCor (white matter and CSF ROIs, 5 components each), scrubbing (as many regressors as identified invalid scans), motion regression (12 regressors: 6 motion parameters + 6 first-order temporal derivatives). A bandpass filter of 0.008–0.09 Hz was applied.

We excluded those participants with mean motion over the scan > 0.3 mm, and those with maximum motion of > 3 mm. 5 participants (2 controls, 2 PD, 1 iRBD) were excluded due to excess motion on fMRI scan.

ROI-to-ROI connectivity of the 10 cerebellar regions, bilateral basal ganglia and thalamic ROIs was determined using Conn, by calculating bivariate Pearson correlation coefficients between all preprocessed ROI timecourses, followed by Fisher’s z transform. The z-transformed data were then analysed at second level using Conn’s data-driven hierarchical clustering method (Nieto-Castanon & Whitfield-Gabrieli, 2022) based on functional similarity metrics and anatomical proximity.

### Statistics

The Conn toolbox was used to perform a 3-group ANCOVA to find any connectivity group differences controlling for participant age. We used the default parametric multivariate statistics (functional network connectivity cluster level inference with cluster threshold *p* < 0.05 and cluster-level p_FDR_<0.05.)

All other statistical analyses were done using R (version 4.3.0). Variables were checked for normality by inspection of histograms and the Shapiro-Wilk test. For normally distributed variables, Fisher’s exact test was used to compare sex between groups, ANOVA was used to compare continuous variables between all three groups, and t-tests between PD and iRBD. T-tests were done without assuming equal variance. The Kruskal-Wallis test was used to compare non normally distributed variables between groups, and the Wilcoxon rank sum test for two group comparisons.

In the Conn ROI-to-ROI ANCOVA analysis, we found one cluster of regional connections which was significantly different between groups. The average connectivity between all the regions in this cluster was calculated for each participant, and correlations were investigated in R (version 4.3.0 https://www.R-project.org/) using linear regression between this average connectivity and clinical scores (UPDRS-III, MoCA) with group, age and sex as covariates.

### Data availability

The data supporting the findings of this study are available on the basis of a formal data sharing agreement and depending upon data usage, agreement for formal collaboration and co-authorship, if appropriate.

## Results

Demographic and clinical characteristics, including motor and cognitive screening scores are reported in Table 1. No significant differences were observed in age and sex between groups. RBDSQ scores were highest in iRBD, and then PD, with all of the iRBD, and 8/17 of the PD having a RBDSQ greater than 5, the cut-off usually used to indicate the presence of RBD based on clinical history.(Nomura et al., 2011) In the iRBD group, 10 participants were taking melatonin, and 3 clonozepam. The amount of motion and other signal change artefact on the fMRI data was not significantly different between groups (see Supplementary Figure S3 and Table S1).

**Table 1:** Demographic characteristics of the participants

	HC [16]	PD [17]	iRBD [13]	Statistics
Age	62.4 (10.9) [46:79]	62.9 (9.5) [47:79]	65.5 (8.1) [51:77]	F_2,43_=0.39 *p* = 0.68
Male	7/16 (43.75%)	12/17 (70.59%)	10/13 (76.92%)	Fisher exact *p* = 0.17
MMSE	29.29 (0.99) [27:30]	28.71 (1.21) [26:30]	28.69 (1.70) [25:30]	χ^2^ = 1.84 *p* = 0.4
MoCA	-	27.00 (2.18) [24:30]	25.54 (2.90) [18:29]	W = 137 *P* = 0.27
MDS-UPDRS-III	-	34.00 (15.52) [15:64]	7.69 (5.98) [1:25]	W = 215 *P* < 0.001
Levodopa (mg)	-	388.24 (265.04) [0.0:900.0]	0.0 (0.0) [0.0:0.0]	-
RBDSQ	1.40 (1.45) [0:4]	5.41 (4.03) [1:13]	9.85 (1.82) [6:13]	W = 40.5 *p* = 0.003
Symptoms duration (months)	-	72.58 (37.91) [15.57:140.07]	66.35 (36.31) [17.57:133.43]	t_26.5_=0.46 *p* = 0.65
Time from PSG to MRI (months)	-	-	42.2 (41.4) [2:116]	-
Years Education (< 12 / 12–13 / 14–17 / 18+)	-	13/1/1/2	7/1/2/3	Fisher exact *P* = 0.57

For Age, Sex, and MMSE the p value represents a comparison over all three groups. Otherwise tests are between PD and iRBD. In the statistics column, F = ANOVA, χ^2^ = Kruskal-Wallis, W = Wilcoxon signed rank, Fisher = Fisher exact test. Values are mean(SD) [range]. MoCA = Montreal Cognitive Assessment, UPDRS-III = Unified Parkinson’s Disease Rating Scale: motor part, MMSE = Mini Mental State Examination, RBDSQ = REM Sleep Behaviour Disorder Screening Questionnaire, PSG = polysomnography

**Fig. 1:**
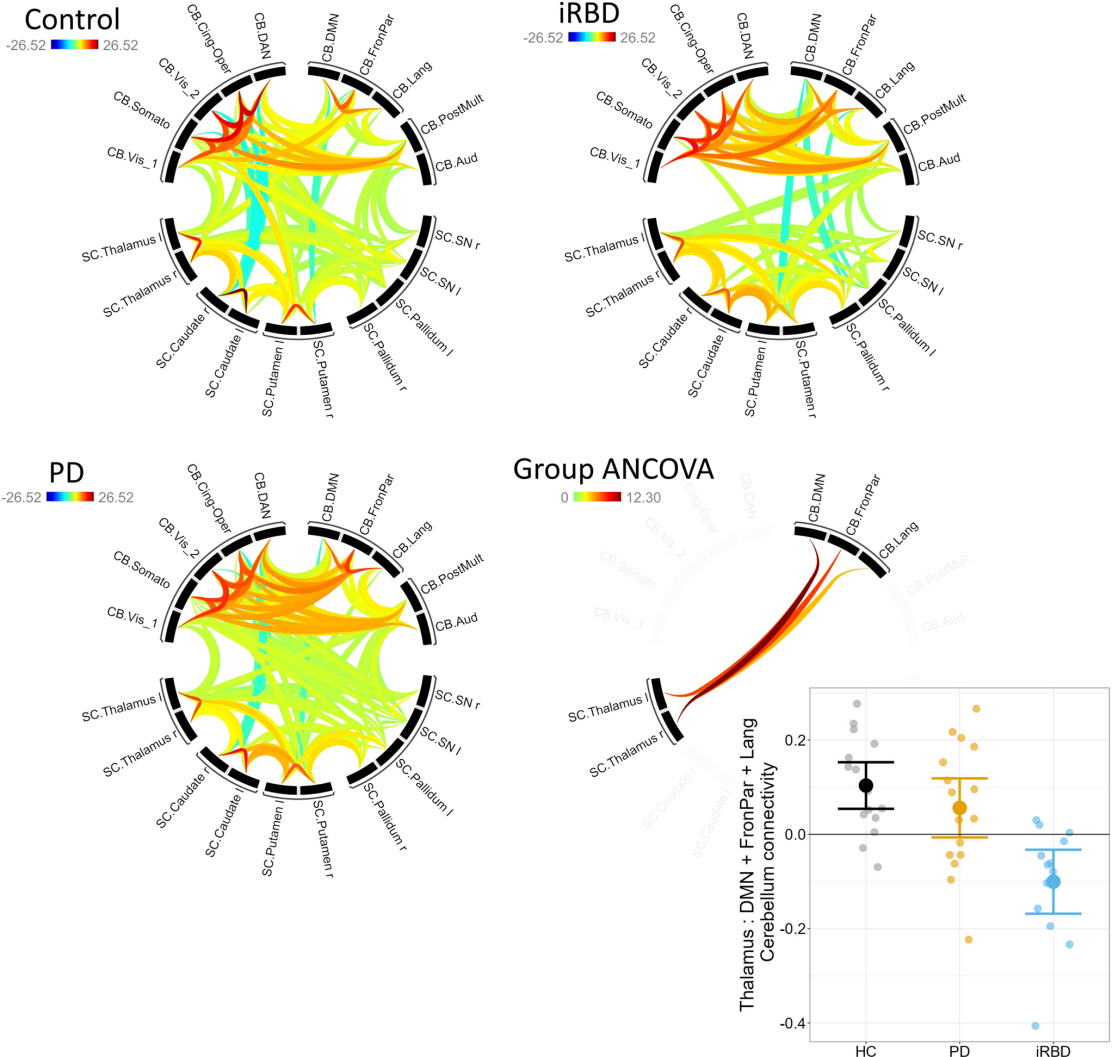
Network connectivity in the basal ganglia, thalamus and cerebellum in the 3 groups, and the overall ANCOVA group difference. The bottom-right graph shows the mean connectivity in the thalamus to cerebellum Language, Frontoparietal and DMN connections (error bars = +- 2SE). Cerebellar networks: Vis_1 = Primary Visual; Vis_2 = Secondary Visual; Somato = Somatomotor; Cing-Op = Cingulo-Opercular; DAN = Dorsal Attention; Lang = Language; FronPar = Frontoparietal; Aud = Auditory; DMN = Default Mode Network; PostMul = Posterior Multimodal. Subcortical regions: Caud = Caudate; SN = substantia nigra; Pall = Pallidum; Put = Putamen; Thal = Thalamus

**Fig. 2:**
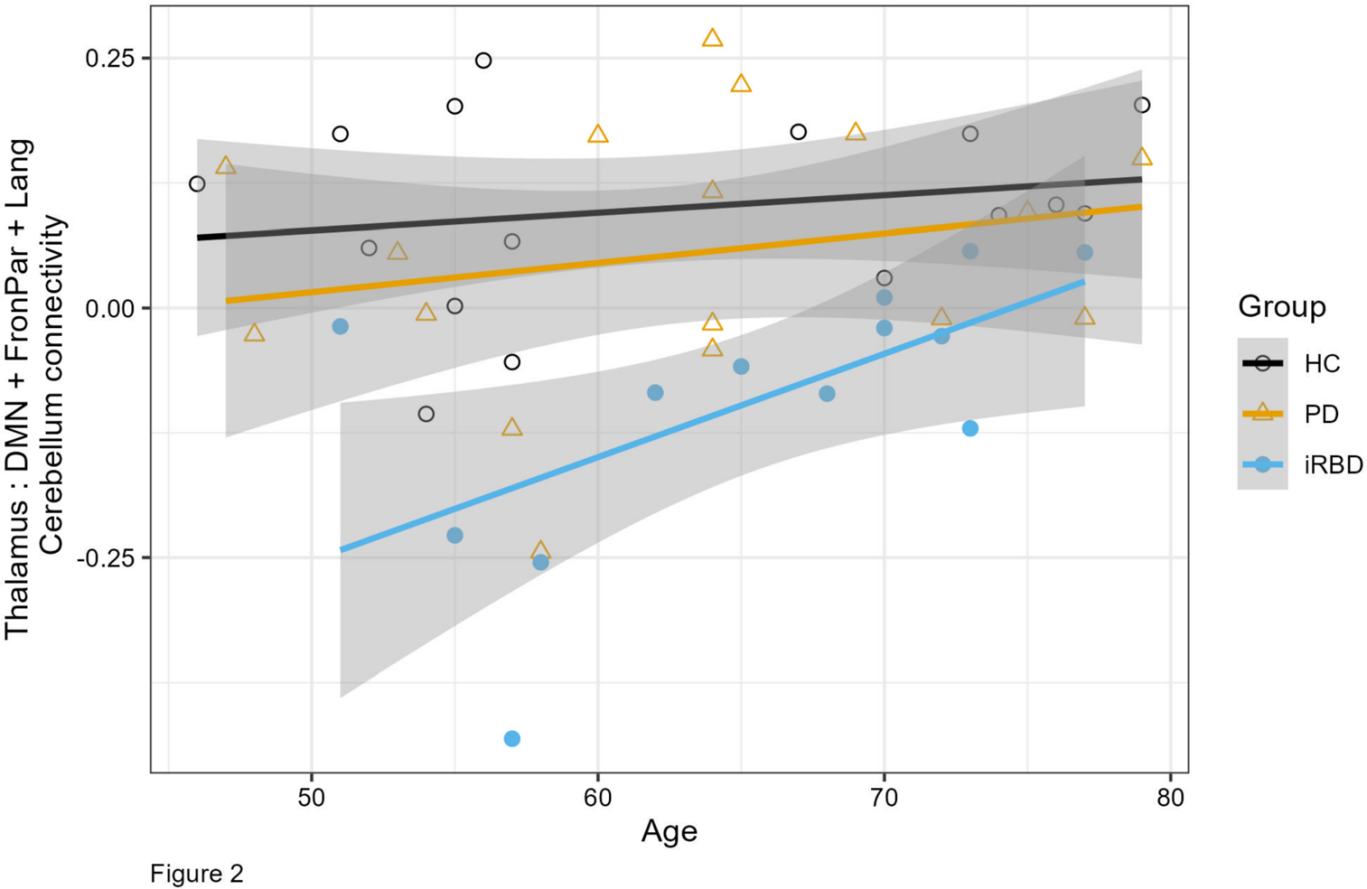
Resting state functional connectivity between the thalamus and cerebellar default mode, frontoparietal and language networks, and age

The connectivity analysis grouped regions according to hierarchical clustering, and identified 7 clusters for the iRBD group (Fig. 1). In the 3-group ANCOVA controlling for age across HC, PD and iRBD, there was a significant difference in connectivity between the thalamus (left and right ROIs) and the cerebellar regions related to the default mode network (DMN), fronto-parietal and language networks (see Fig. 1), F_4,82_ = 5.47, p(FDR corrected) = 0.017. Post-hoc analysis (line graph on Fig. 1) revealed a reduction in connectivity in the iRBD group compared to controls (Tukey post hoc *p* < 0.001) and PD (Tukey post hoc *p* = 0.002). No other connectivity differences were found among the investigated regions. Therefore, the mean connectivity value for this cerebellar functional network (thalamus to cerebellar regions relating to DMN, fronto-parietal and language networks) was calculated, and its association with clinical variables was investigated. Linear regression showed that there was a positive association between this cerebellar functional network connectivity and age (beta = 0.0039 CI = 7e-05 : 0.0077, *p* = 0.046; Fig. 2) but not sex (*p* = 0.7). There was no significant association in the PD group with levodopa dosage (*p* = 0.8), nor was there a significant difference in the cerebellar functional network between PD scoring above 5 on the RBDSQ vs. those with a lower score (*p* = 0.5).

We investigated associations between MoCA, UPDRS-III in the combined iRBD and PD group with covariates of age, sex and group. MoCA and UPDRS-III did not have a significant relationship with the connectivity measure.

## Discussion

We found evidence of reduced connectivity between the thalamus and cerebellar functional network in the iRBD group compared to both PD and healthy control participants. Specifically, affected cerebellar regions were related to the default mode, language and frontoparietal resting-state networks. The mean PD connectivity was intermediate between HC and iRBD, but not significantly different from HC.

Since both iRBD and PD show many motor and non-motor manifestations that are not under voluntary control, investigating the connectivity between subcortical structures (e.g. basal ganglia, thalamus, cerebellum) may reveal dysfunctions that are important in the manifestation of such symptoms. Recent in vivo studies in iRBD (Knudsen et al., 2018) show an ascending degree of abnormalities (from the gut to the brainstem) that may not as yet involve cortical structures. So cortical connectivity abnormalities may actually be driven by abnormally functioning subcortical structures.

To the best of the authors’ knowledge, only a few previous studies investigated connectivity between the cerebellum and subcortical nuclei, especially the thalamus and basal ganglia. However, subcortical structures’ connectivity could be altered in synucleinopathies, as these initially involve subcortical nuclei. Some studies have investigated connectivity between cortical areas and either the cerebellum or other subcortical grey matter structures in iRBD with resting state fMRI. Yamada et al. (2019) found increased connectivity between cerebellum and somatosensory cerebral cortex in those with iRBD and mild motor impairment vs. no motor impairment, but did not find cerebellar-to-cortical or cerebellar-to-striatal differences between iRBD either with or without motor impairment compared to healthy controls. Although this finding of increased connectivity in cerebellar-cortical connectivity may seem at odds with our finding of reduced cerebellar-thalamic connectivity, it should be noted that they employed a cerebellar anatomical atlas, rather than a functional one, therefore the regions involved in disrupted connectivity may be different in their study compared to ours. Li et al. (2021) found reduced connectivity in iRBD between a whole-brainstem ROI and bilateral clusters in the cerebellum, located in the frontoparietal resting state network. Byun et al. (2020) investigated connectivity to a thalamus ROI in iRBD and found increased connectivity with the occipital lobe in iRBD, but did not report relationships with the cerebellum. In a study comparing PD with vs. without iRBD, (Liu et al., 2021) increased fMRI Regional Homogeneity (a measure of localised synchronous activity) was found in the cerebellum of PD-RBD (in the somatomotor network) with increased connectivity between this region and occipital, temporal and supplementary motor areas. Although these studies are conceptually related to our findings, it is difficult to directly compare their results with our own, due to differences in methodology and regions used.

Our finding of reduced cerebellar-thalamic connectivity in the default mode, language and frontoparietal resting-state networks shows mechanistic subcortical involvement that may underlie the known cortical connectivity abnormalities. Cognitive decline is common in iRBD and PD with RBD, with the domains of attention/executive function and language often affected (Maggi et al., 2021; Lin & Chen, 2018). The frontoparietal network is involved in cognitive control and modulating alertness (Menon & D’Esposito, 2022) with changes in the network after sleep deprivation correlating with attentional performance. (Yao et al., 2023) Hence the changes to the connectivity of the language and frontoparietal network regions of the cerebellum may relate to ongoing decline in these functions, and future investigation of the association of cerebellar connectivity with specific language and attentional symptoms would help understand the mechanism behind these symptoms, and hence aid in developing treatment.

We found greater evidence of changed connectivity in the iRBD participants compared to the PD group (Fig. 1). A number of reports have found abnormalities in the thalamus and cerebellum which are related to the presence of RBD in PD, with reduced volume of the frontoparietal network area of the cerebellum, (Boucetta et al., 2016) and of the thalamus (Boucetta et al., 2016; Oltra et al., 2022) in those with PD with vs. without RBD. The RBDSQ was also found to negatively correlate with thalamus volume in PD (Kamps et al., 2019), and reduced local network efficiency throughout the cerebellum (Guo et al., 2018) has been seen in PD with vs. without RBD. It is thus possible that the cerebellar-thalamic connectivity changes that we find are specific to the RBD pathophysiology. In our participants, the RBDSQ was significantly higher in iRBD vs. PD, indicating that there were fewer symptoms of RBD in the PD group. Hence, if the cerebellar-thalamic connectivity changes are specific to RBD pathophysiology, the altered connectivity in the iRBD vs. PD could be due to lower frequency of RBD symptoms in our PD participants.

Disease heterogeneity in PD may also play a role in the connectivity differences between iRBD and PD. Although the timespan to motor phenoconversion in iRBD is highly variable, its pathological progression is thought to be predictable. (Fedorova et al., 2022; Knudsen et al., 2018) Conversely, PD is a much more heterogeneous condition in terms of brain topography progression; some may have an ascending pathological gradient, while others may have a descending gradient (Horsager et al., 2020). The heterogeneity in pathological involvement may be the cause of these findings in our iRBD and PD cohorts. Furthermore, additional modifications of the connectivity based on the type and degree of the predominant motor manifestation in PD may also happen. Reports of cerebellar-thalamus connectivity have been mixed in PD, with Kawabata et al. (2020) reporting decreased connectivity, Chen et al. (2023) reporting a decrease in connectivity to the left thalamus in the tremor dominant, but not postural instability gait dominant group, vs. Zhang et al. (2016) who found increased connectivity in patients with tremor compared those without. Hou et al. (2018) did not find any difference from healthy control, and Choi et al. (2022) found increased connectivity in DLB with parkinsonism vs. those without and healthy controls.

Yamada et al. (2019) found higher cortico-cerebellar connectivity in iRBD with vs. without mild motor impairment. Possibly, the initial dysfunction of brainstem nuclei which causes the lack of sleep atonia leads to altered cerebellar input, decreasing the cerebellar connectivity, but as the pathology progresses and affects other brain regions, then the inputs to the cerebellum further change causing a shift in cerebellar-thalamus connectivity. Future work could investigate changes in connectivity as RBD progresses.

We did not observe any associations between cerebellar functional network connectivity and levodopa dose. However, it is possible that since all PD participants were on individually optimised doses, and scanned on medication, this may have modified connectivity, perhaps with a ‘normalisation’ effect. A recent report (Liu et al., 2024) found decreased thalamus-cerebellum connectivity in PD off medication vs. controls, but no differences on medication. It is thus possible that the decreased connectivity in iRBD vs. PD is due to the latter being scanned on medication.

We observed a significant positive association of age with the cerebellar functional network connectivity. A large recent study of functional connectivity found a positive association between aging and connectivity between the thalamus and regions in the cerebellar DMN and frontoparietal networks during movie watching. (Wang et al., 2023) However, they did not find significant associations during resting state. Our iRBD participants were slightly (though not significantly) older than the other groups, but since we found a decrease in the connectivity in iRBD, their older age would have tended to diminish group differences.

Several limitations of our study should be addressed. The number of participants in each cohort is small, therefore our exploratory analysis should be confirmed in future studies. Furthermore, given the exploratory nature of the study, we may have been underpowered to detect subtle differences and associations in and across groups. We did not include any participants with dementia with Lewy bodies (DLB), which has RBD as a core clinical feature. This limits our ability to infer the relationship between our connectivity changes in iRBD and development of a predominantly cognitive vs. motor phenotype. Only iRBD patients underwent video-polysomnography, therefore findings relating RBD symptoms and PD should be interpreted with caution. One iRBD had an MDS-UPDRS-III score of 25 (all other iRBD were < 13; see Table 1). However, upon examination and subsequent re-evaluation of this participant’s clinical characteristics by a movement disorders expert (N.P.) these features were judged to be possibly related to other comorbidities. Ten points were related to presence of mild resting, postural and intention tremor, which was persistent during the examination. The patient did not fulfil the clinical criteria for Parkinson’s and the DaTSCAN findings did not support a diagnosis of PD. We have repeated our analysis after excluding this participant (see Supplementary Table S2, Figures S4 and S5) and our results are essentially unchanged, with the same cerebellum-thalamus connectivity identified as significantly different in the iRBD group. The iRBD group were taking either melatonin or clonazepam, and it is possible that these drugs may have altered their functional connectivity. However, we are unable to comment on the effect these drugs might have had on connectivity in this study due the lack of previous studies. The PD group were taking dopaminergic medication and were scanned in the ON state, whereas none of the iRBD group were taking such medication. Dopamine replacement in PD would act to normalise basal ganglia–frontal and DMN connectivity and has been reported to increase thalamic self-inhibition. (Dirkx et al., 2017) However, this would be expected to decrease the influence of the cerebellum on the thalamus activity contrary to our finding of greater connectivity in PD vs. iRBD, suggesting that medication may not be the reason for the relatively normal cerebellar–thalamus connectivity in PD. Cognitive investigations were limited to screening tests, i.e. the MoCA: it should be acknowledged that a more refined cognitive evaluation may uncover more pathophysiological relationships.

## Conclusions

In conclusion, compared to PD and HC, in iRBD we found reduced connectivity between the thalamus and regions of the cerebellum involved in the default mode, frontoparietal and language networks. The association between the burden of RBD symptoms and connectivity in cerebellar-thalamic networks deserves further investigation.

Longitudinal studies may help understand how connectivity progresses in relation to symptoms development, or whether it has any prognostic value regarding conversion to PD, DLB or MSA.

## Electronic supplementary material

Below is the link to the electronic supplementary material.

Supplementary Material 1

## Data Availability

The data supporting the findings of this study are available on the basis of a formal data sharing agreement and depending upon data usage, agreement for formal collaboration and co-authorship, if appropriate.
