# Supplementary material for: Cerebellum and basal ganglia connectivity in isolated REM sleep behaviour disorder and Parkinson’s disease: an exploratory study
Source: Brain Imaging Behav. 2024 Sep 25;18(6):1428–37. doi: 10.1007/s11682-024-00939-x (PMC11680622; doi:10.1007/s11682-024-00939-x)

Table S1 fMRI quality check metrics from CONN software for each group.

|  | HC [18] | PD [19] | iRBD [14] | Stats |
| --- | --- | --- | --- | --- |
| QC_MeanMotion | 0.17 (0.072) [0.071:0.37] | 0.20 (0.078) [0.09:0.43] | 0.17 (0.059) [0.091:0.35] | F_2,48_=0.73 p=0.49 |
| QC_MaxMotion | 1.32 (1.27) [0.18:5.69] | 1.21 (1.00) [0.31:4.36] | 0.86 (0.54) [0.23:1.94] | F_2,48_=0.85 p=0.44 |
| QC_InvalidScans | 6.28 (8.48)  [0 : 26] | 5.68 (9.57)  [0 : 36] | 2.07 (2.37)  [0 : 8] | F_2,48_=1.29 p=0.29 |
| QC_MeanGSchange | 0.79 (0.05) [0.73:0.95] | 0.79 (0.055) [0.68:0.86] | 0.79 (0.049) [0.70:0.86] | F_2,48_=0.0076 p=0.99 |

Table S2

Table 1 Demographic characteristics of the participants excluding the iRBD participant with UPDRS-III = 25

|  | HC [16] | PD [17] | iRBD [13] | Statistics |
| --- | --- | --- | --- | --- |
| Age | 62.4 (10.9) [46 : 79] | 62.9 ( 9.5)  [47 : 79] | 64.5 (7.7) [51.0:73.0] | F_2,42_=0.17 p=0.85 |
| Male | 7/16 (43.75%) | 12/17 (70.59%) | 10/12 (83.33%) | p=0.088 |
| MMSE | 29.29 (0.99) [27 : 30] | 28.71 (1.21)  [26 : 30] | 28.92 (1.56)  [25 : s30] | χ^2^ = 1.97  p=0.4 |
| MoCA | - | 27.00 (2.18)  [24 : 30] | 26.17 (1.90)  [22 : 29] | W = 120  P = 0.4 |
| MDS-UPDRS-III | - | 34.00 (15.52) [15 : 64] | 6.25 (3.08)  [1 : 12] | W=204  P<0.001 |
| Levodopa (mg) | - | 388.24 (265.04)  [0.0 : 900.0] | 0.00 (0.00) [0.00:0.00] | - |
| RBDSQ | 1.40 (1.45) [0 : 4] | 5.41 (4.03)  [1 : 13] | 9.83 (1.90) [6:13] | W = 38  P=0.005 |
| Symptoms duration (months) | - | 72.58 (37.91) [15.57:140.07] | 60.76 (31.55) [17.57:109.13] | t_26.2_=0.91 p=0.37 |
| Time from PSG to MRI  (months) | - | - | 36.8 (38.4)  [2 : 116] | - |
| Years Education  ( <12 /12-13/14-17 / 18+ ) | - | 13/1/1/2 | 6/1/2/3 | Fisher exact  P =0.52 |

Figure S1A The cerebellar networks overlaid on the cerebellar template
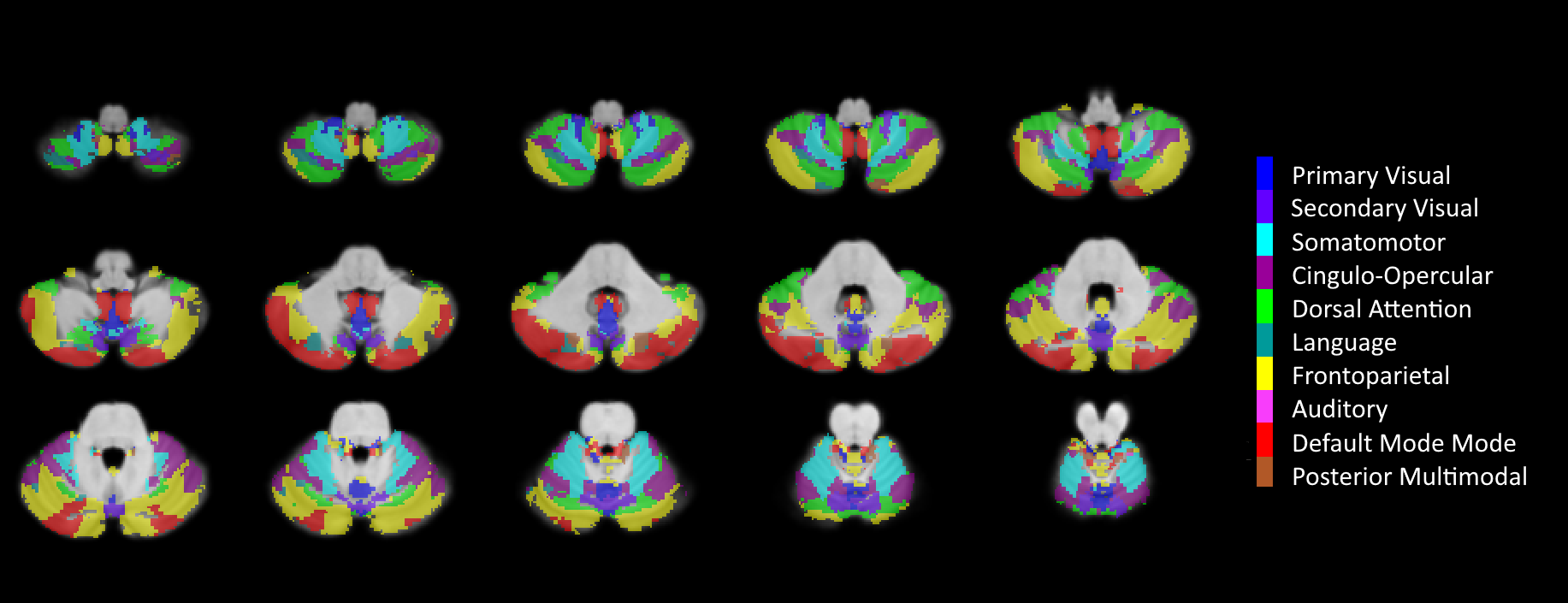


Figure S1B The cerebellar networks overlaid on a typical iRBD subject’s fMRI scan


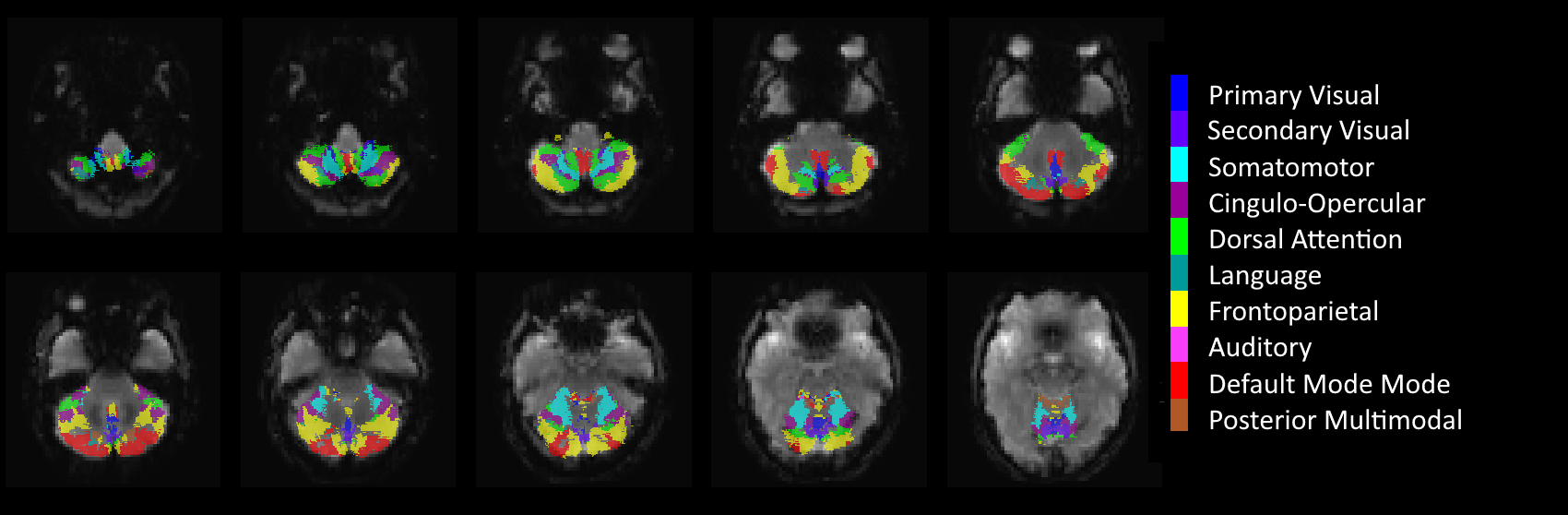


Figure S2 Subcortical regions overlaid on a typical iRBD subject’s structural scan


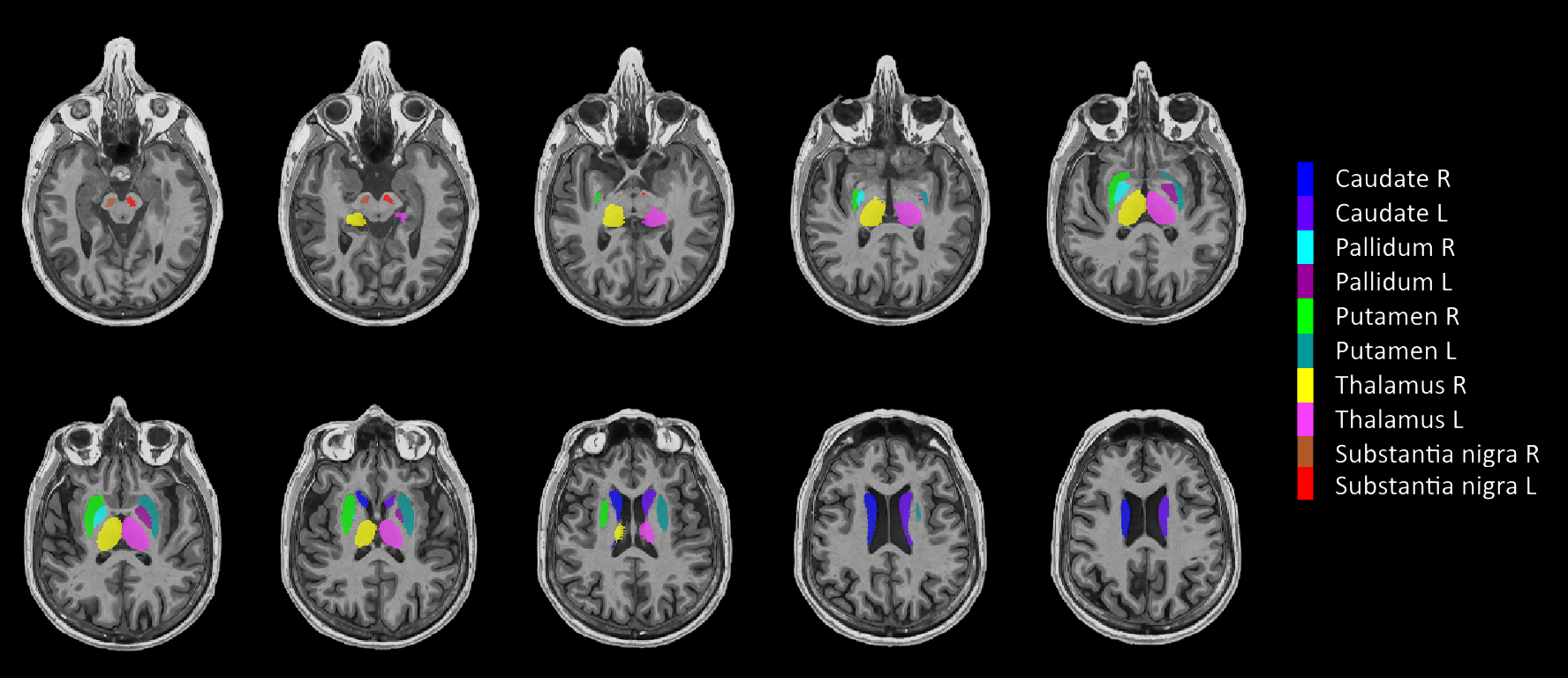


Figure S3 fMRI quality check metrics from CONN software for each group.


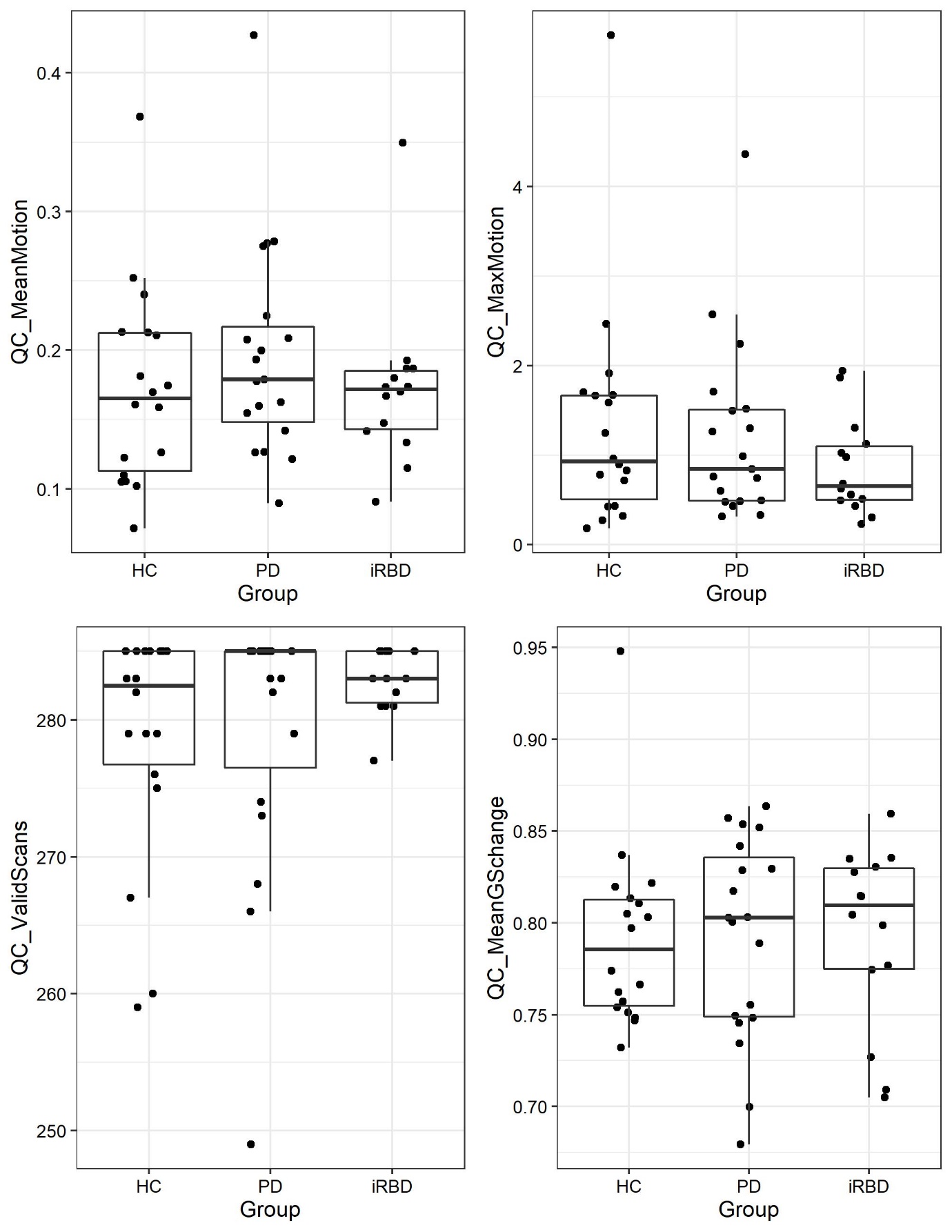


Figure S4 Excluding the iRBD participant with high UPDRS-III score; between group network connectivity A) ANOVA Network connectivity group difference [ F(4,80) = 5.58, p-FDR = 0.0144] B) Mean connectivity in the thalamus to cerebellum Language, Frontoparietal and DMN connections (error bars = +- 2SE). iRBD < HC (Tukey post hoc p <0.001); iRBD < PD (Tukey post hoc p =0.0017)


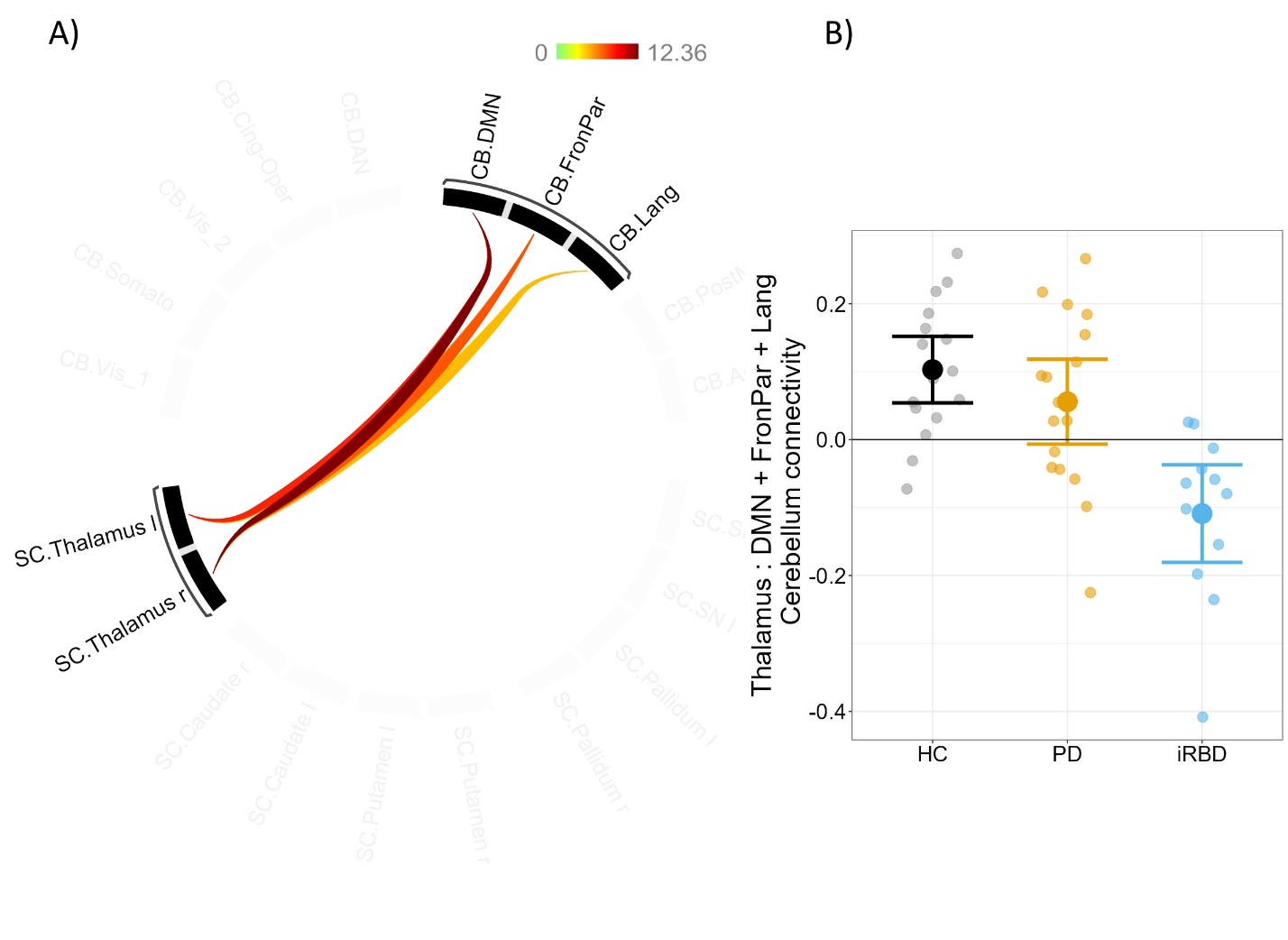


Cerebellar networks: Vis_1 = Primary Visual; Vis_2 = Secondary Visual; Somato = Somatomotor; Cing-Op = Cingulo-Opercular; DAN = Dorsal Attention; Lang = Language; FronPar = Frontoparietal; Aud = Auditory; DMN = Default Mode Network; PostMul = Posterior Multimodal. Subcortical regions: Caud = Caudate; SN = substantia nigra; Pall = Pallidum; Put = Putamen; Thal = Thalamus

Figure S5 Excluding the iRBD participant with high UPDRS-III score; Resting state functional connectivity between the thalamus and cerebellar default mode, frontoparietal and language networks, and age (Age beta coefficient 0.0035 ; p = 0.077)


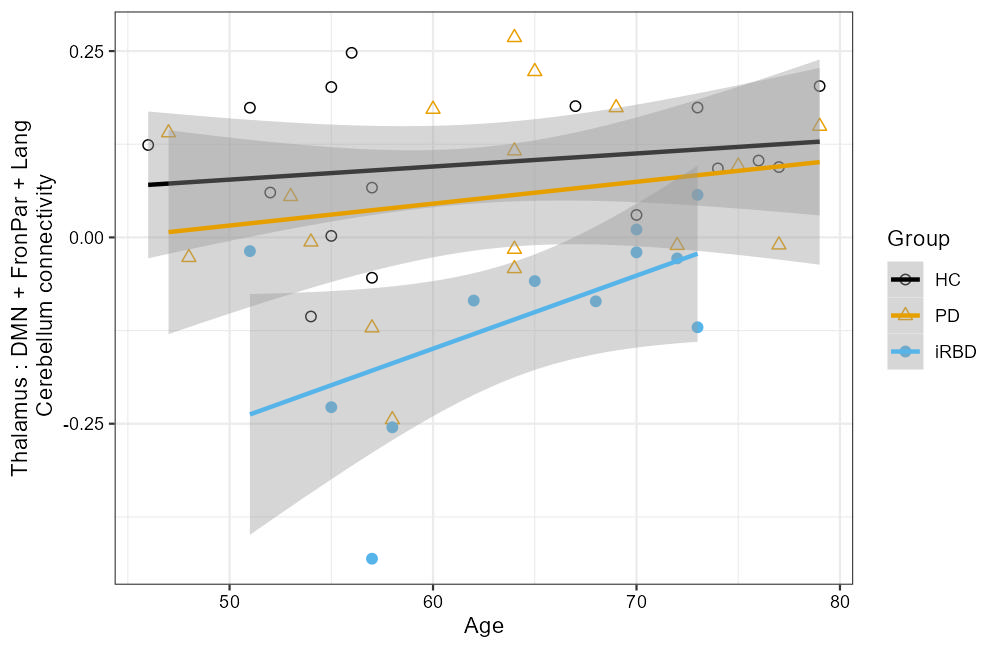

Supplement: Supplementary file 1 — Supplementary Material 1 [file 11682_2024_939_MOESM1_ESM.docx]
